# Home-dwelling persons with dementia’s perception on care support: Qualitative study

**DOI:** 10.1177/0969733019893098

**Published:** 2020-01-27

**Authors:** Stein Erik Fæø, Frøydis Kristine Bruvik, Oscar Tranvåg, Bettina S Husebo

**Affiliations:** University of Bergen, Norway; University of Bergen, Norway; University of Bergen, Norway; Western Norway University of Applied Sciences, Norway; Oslo University Hospital, Norway; University of Bergen, Norway

**Keywords:** Assistive technology, decision making, dementia, hermeneutics, patient participation, volunteer support, care support, ethics

## Abstract

**Background:**

Over the last years, there has been a growth in care solutions aiming to support home-dwelling persons with dementia. Assistive technology and voluntarism have emerged as supplements to traditional homecare and daycare centers. However, patient participation is often lacking in decision-making processes, undermining ethical principles and basic human rights.

**Research objective:**

This study explores the perceptions of persons with dementia toward assistive technology, volunteer support, homecare services, and daycare centers.

**Research design:**

A hermeneutical approach was chosen for this study, using a semi-structured interview guide to allow for interviews in the form of open conversations.

**Participants and research context:**

Twelve home-dwelling persons with dementia participated in the study. The participants were recruited through municipal daycare centers.

**Ethical considerations:**

Interviews were facilitated within a safe environment, carefully conducted to safeguard the participants’ integrity. The Regional Committee for Medical and Health Research Ethics, Western Norway (Project number 2016/1630) approved the study.

**Findings:**

The participants shared a well of reflections on experience and attitudes toward the aspects explored. They described assistive technology as possibly beneficial, but pointed to several non-beneficial side effects. Likewise, they were hesitant toward volunteer support, depending on how this might fit their individual preferences. Homecare services were perceived as a necessary means of care, its benefits ascribed to a variety of aspects. Similarly, the participants’ assessments of daycare centers relied on specific aspects, with high individual variety.

**Discussion and conclusion:**

The study indicates that the margins between whether these specific care interventions were perceived as supportive or infringing may be small and details may have great effect on the persons’ everyday life. This indicates that patient participation in decision-making processes for this group is—in addition to be a judicial and ethical requirement—crucial to ensure adequate care and support.

## Introduction

The coming years will see a rapid increase in the number of persons with dementia.^1^ The World Health Organization has labeled dementia a global public health priority and a growing number of nations are developing dedicated plans on how to provide adequate care and support for persons with dementia and their carers.^2^ To meet these challenges, there has been a substantial growth in care solutions aiming to enable persons with dementia to live better and safer at home. Within governmental dementia plans, especially innovative assistive technology and increased emphasis on the potential within volunteer support have been defined as areas of commitment alongside the traditional homecare and daycare services.^3,4^

Various kinds of assistive technology have seen a massive growth during the past few years. These ranges from traditional safety alarms and stove guards to the more recent electronic pill dispensers, robots, and advanced sensor and monitoring technologies.^5,6^ However, although perceived as useful for caregivers, technology that directly helps the person with dementia has proved challenging to implement,^7^ and patients often face pressure on when and where to use such equipment.^6^ In addition, to uphold social interaction and prevent isolation, there has been a growing emphasis on the use of volunteer support within dementia care.^3,4^ However, there is limited research within this field and what exist indicates that it is challenging to optimize this kind of support.^8,9^ As a baseline of care, access to homecare services, such as support in medication, personal hygiene, and nutrition, has been a clear demand from this group.^10^ Alongside these services, daycare centers, providing meaningful activities is increasingly common, and research indicates several benefits for people living with dementia.^11–13^

Despite growing attention to new ways of thinking care and support for this group, evidence on what works to provide appropriate support is ambiguous. In a large systematic review, Dawson et al.^14^ reveals that intervention studies in general show little or no effect on most outcomes, but highlight individualized and flexible care solutions as success factors. These findings are in line with the emphasis of individualized and person-centered care for persons with dementia as this concept has evolved for almost 30 years.^15^ Still, insufficient acknowledgment of the complexity of the field and lack of knowledge of the perspectives of persons with dementia and their informal caregivers are described as weaknesses in the evidence base.^14^ Lack of patient participation is widely reported within dementia care and persons with dementia experience exclusion from decision-making processes considering their own care often without proper assessment of their capacity to such participation.^16,17^ Often, family caregivers or healthcare personnel make these decisions *for* the patient, even when the person with dementia has expressed alternative wishes. However, Elliott et al.^18^ state that knowing the person’s life story and planning enable caregivers to make choices in line with the person’s wishes. As depending on help from others in itself poses as a threat of feeling inferior as a human being,^19^ being left out from decision making further constitutes a threat of marginalization and exclusion for persons with dementia.^20^ The patients’ right to participate in these kinds of processes are considered a basic human right and the United Nations has criticized the denying of these rights based on diagnosis or standardized assessments of mental capacity. Rather, healthcare personnel are instructed to support the person to exercise their legal capacity in this matter.^21,22^ This underscores the need for more knowledge about how home-dwelling persons with dementia perceive the support measures offered, how these care solutions influence their everyday life, and how to arrange for optimized patient participation within dementia care.

### Aim

The aim of this study was to explore and describe the perceptions of home-dwelling persons with dementia on assistive technology, volunteer support, homecare services, and daycare centers. The following research questions were addressed: (1) How do persons with dementia describe their present and past experiences related to assistive technology, volunteer support, homecare services, and daycare centers? and (2) How do persons with dementia describe their attitudes toward receiving these support measures in the time to come? The overarching purpose was to increase our knowledge of the own perceptions of persons with dementia when given the opportunity to express their personal views.

## Methods

A qualitative, exploratory design, based upon hermeneutical methodology was chosen for this study.

### Participants and recruitment

Four daycare centers for persons with dementia helped recruit participants after the following inclusion criteria: having a registered dementia diagnosis according to the ICD-10 criteria^23^ in their electronic journal, aged 65 years or above, living at home, and able and willing to consent to and participate in an interview conversation. To reduce sharing of sensitive patient data, healthcare personnel at the daycare centers assessed fulfillment of inclusion criteria. They also assessed ability to consent and participate based on an overall assessment of the participants’ ability to comprehend and judge the potential risks and benefits of participation in accordance with the Norwegian Patients’ Rights Act^24^ and the Helsinki Declaration.^25^ Thirteen people consented to participate in this study. Among these, one person withdrew due to an acute incident. The participants, six women and six men, were aged between 69 and 89 years old. Six of them were living alone and six were receiving homecare. All participants attended a daycare center and this was the site for recruitment to the study.

### Data collection

The hermeneutical methodology rests upon the claim that all understanding and knowledge founds upon context and tradition.^26^ This means that the researcher’s pre-understanding, based on their own background and experience, will influence the study. This implies a need to reflect on how this pre-understanding affects all parts of the study, from design through conduction as well as during interpretation of the empirical data collected. The researchers in this study had clinical experience with care work for persons with dementia within homecare, nursing homes, hospitals, or psychiatric wards, as a nurse or medical doctor. When communicating with persons with dementia, open questions is recommended to safeguard the others’ integrity.^27^ Likewise, an open approach in the meeting is recommended within the hermeneutical tradition.^26^ Therefore, an open and flexible interview guide was developed to give the interviews a form of open conversations, more than sessions of questions and answers. The interview conversations opened by letting the participants tell about themselves and exploring their perceptions and attitudes toward living at home, at the present time and in the future. Findings from this part of the interviews are published elsewhere—and focus on the significance of the home as such, as described by the participants.^28^ The interviewer introduced the themes assistive technology, volunteer support, homecare, and daycare centers where this was natural in the conversation, to explore their own experience and attitudes. Initially, the participants often expressed difficulties in reflecting on measures they had no direct experiences with, but as the conversations evolved and they described challenges in everyday life, we came back to how different measures could help. Most often, they would then give comprehensive reflections on their attitudes toward these suggestions. When given time, space, and explanation, all participants shared substantial reflections on the themes of inquiry. All interviews were conducted either at the participants’ home or at the daycare centers, at the wish of the participants.

### Analysis and interpretation

All interviews were transcribed verbatim successively, followed by initial analysis of each interview as a single text. After the first two interviews, two of the authors (S.E.F. and O.T.) analyzed the texts to search for unexpected themes to follow up in further interviews and to critically examine the lapse of the interviews. This process was repeated with all researchers participating after five interviews. After all interviews were transcribed, two of the authors (S.E.F. and O.T.) analyzed each interview as single texts, first separately, then in dialogue. Thereafter, the whole research group repeated this process. All texts were then explored as a whole, increasing our understanding of each interview, as well as of the entire text as a whole. Underway, we discussed various theoretical frameworks and how these could potentially lead to additional understanding of the empirical data. In line with hermeneutical principles for interpretive understanding, this circular interpretive movement between the individual texts and the text as a whole characterized the analytical process, which lasted throughout the writing process.^29^

### Ethics

Eligible participants were provided with written and oral information about the study by healthcare personnel at the daycare centers, and all participants gave written consent to participate. Information on the purpose of the interview, recording, and data treatment were repeated immediately before every interview along with information on the right to withdraw at any time, without any consequences. Issues of moral sensitivity^27^ were highlighted and discussed while preparing the study, and the interviewer paid close attention to the participants’ reactions during the interview to avoid distress. Wordings and degree of probing were constantly adjusted to make sure that the participant felt comfortable in the situation. Some participants also had a family caregiver present during the whole or parts of the interviews. Healthcare personnel were encouraged to follow up the participant after the interview. Signs of unease after the interviews were reported in a few participants, but most reported feedbacks of the interviews as a positive experience. All data have been de-identified and are confidentially treated. The study was approved by The Regional Committee for Medical and Health Research Ethics, Western Norway (Project number 2016/1630).

## Results

### Assistive technology—safety with side effects

The participants expressed few experiences with or knowledge about assistive technology. Two participants had a stove guard and one had a safety alarm. The participant having a safety alarm expressed how this made her feel safe at home: “I think it’s safe and good to have. But I have never used it. No, that’s why I use it, (laughs)” (woman, 89). Although she had never needed to use the alarm, she put it to good use as a safety measure, knowing that she could get in touch with help in a case of emergency. The feeling of safety was also emphasized by one participant, considering the stove guard:

I’ve got one of these…blinking on the wall, if I forget (the stove). Yeah, it turns off…But I can hear it at once, you know, and then I get the shivers…Oh, it’s creepy! (Woman, 82)

The sound of the stove guard made her shudder at the thought of what might have happened. Knowing it would turn off the stove should she forget made her feel safe. However, the other participant with a stove guard had quite a different perception of how this affected her everyday life:

No, now I won’t be doing any more baking. I’ve got something called “Anna” on the stove because…It ruined the whole stove. It turns on and off at its own will. Suddenly it starts howling and a red light appears…It was the most foolish thing ever done…It was done with good intentions; it was the best to me. But I don’t think so.…(Woman, 87)

Her lacking competency in handling the stove guard had removed her opportunity to bake, and baking had been a meaningful activity for her. She understood why she had been given the device, but did not perceive its benefit as outweighing the side effect—namely, to be unable to handle the stove. In general, the participants’ ability to handle technological equipment was an essential aspect in their attitudes toward these measures. One participant had an initial attitude that receiving assistive technology sounded “despairing.” However, after having some possibilities explained to him, he changed attitudes slightly, but with a condition: “I guess it could be okay, but inherently, I can’t handle it myself” (man, 83). The participant admitted the possibility that assistive technology might be beneficial, but he was also aware that he would not be able to learn how to operate new equipment.

Most participants shared this initial hesitation toward assistive technology but became more positive as possibilities were discussed. Still, most of them expressed some form of reservations. One woman was quite positive to the possibility of receiving an electronic pill dispenser because she had a wish to reduce the number of visits from homecare services. However, her attitude changed when she was told that the device would “beep” to indicate when it was time to take the medicines: “No, I don’t want that beeping!” (woman, 82). The remark was followed by a story about how beeping alarms and the likes would confuse and agitate her. She was adamant that the positive aspects of such a device could not outweigh her antipathy toward its side effects.

### Volunteer support—the complexity of preferences

Only one participant had direct experience with volunteer support. She had been assigned a volunteer to help her keep up her interest in going for walks. However, the arrangement did not work out as she had hoped:

Yes, a lady came and talked…and talked and talked, (laughs). It was nice, you know, but there was no walking…because she just wanted to sit and talk. (Woman, 82)

We cannot know the intention behind this first meeting. Nevertheless, the woman had expected to go for a walk and was disappointed. She was able to laugh about it, but the quote reveals a vulnerability in building expectations on how support should function. Another participant described how he wanted things to be, as the conversation turned to the theme:

You know, there are a lot of single people who would have liked to have someone to talk to…We could be together, two or three persons; there is room for that, both in the living room and on the patio. (Man, 87)

This man, who lived relatively isolated, clearly envisioned how he and the volunteer(s) might benefit from getting together. However, similar to the hesitant attitude toward new and unknown technological devices, the participants also demonstrated the same attitude toward receiving volunteer support. As the conversations touched on activities that the participants no longer were practicing due to declining health, the interviewer would suggest a volunteer as a possible way to keep up these activities. One participant was positive toward having a volunteer in order to be more active, but having a volunteer to help him keep up the activity he loved most was out of the question:

I’m not going hunting without a gun and not being able to shoot. Just tag along with someone who’s hunting, that’s…(Laughs), no! I’ve been hunting so much that…If I’m going hunting, it is me who is going hunting…I learned that from my father, a very good hunter. There was nothing social (about hunting). Hunting, that was one man against…(laughs). (Man, 69)

Hunting was a highly valued activity for this participant, connected with strong emotions involving what his father had taught him. These emotions associated with hunting made the proposal of partaking in a light version of hunting, following a volunteer hunter, almost a personal affront. This reveals that individualization may require more than simply asking about preferences and interests.

### Homecare services—the diversity of care experience

Considering homecare, the participants had differing experience. Most of those living alone had homecare services for supervision of medicine use and nutrition. Most were happy with this arrangement, although they perceived the visits as being short and task oriented:

You know, someone comes to me in the morning, to give me those pills. They chatter every time they see me, you know: “Have you been eating today? Have you had breakfast?”…It’s good for us to have people like that. They watch over us.…You know, they’re just stopping by, and then they have to fly off…But of course they are very nice and cheerful and smiling. So it’s cozy, it is. (Woman, 82)

For this woman, the sense of being seen and taken care of seemed to be the primary perceived benefit of homecare. Even though the visits were short, these comforting and cheerful meetings seemed to brighten up her day. Another female participant, who was anxious about whether she had taken her medicines or not, emphasized the safety in having this taken care of for her.

The home care service comes and delivers them (the pills) and sees to it that I take them, ’cause I’m so afraid that if they don’t watch me…I can’t be sure that I’ve taken them. (Woman, 87)

Knowing she would get the right medicines at the right time made her express a feeling of safety while living alone. Thus, although both women got mostly the same kind of support and were largely equally satisfied, they based their gratification on quite different aspects of the support. Among participants not receiving homecare, most were glad to manage without this support. All the while, they were aware that it might be necessary to accept such support in the future:

For the time being, I’ve been able to manage on my own, but of course it might be relevant…If I need more help, I might be lucky and get something…I sort of feel that it’s best to ignore it. Because now I know nothing about how it’s gonna be, and then I guess it’s important to enjoy the time one has left. (Woman, 86)

The participant expressed a wish to cope at home without support as long as possible and tried not to think about having to accept homecare support at some point in the future. Presently, the main focus was on enjoying the here-and-now, not worrying too much about the future. At the same time, the participant realized this possibility and hoped the help would be available when needed—a perspective shared by most of the study participants.

### Daycare centers—it’s all in the details

All participants had thoughts on the respective daycare centers they were attending. While some had attended their daycare center for several years, others had just started. Likewise, the frequency of attendance varied from 1 to 4 days a week. Most had a positive view of these centers as a place that offered them the opportunity of broaden their everyday environment, as well as sharing meals and activities with others. A woman described how the personnel of the daycare center gave her a personal invitation while she was having an unpleasant stay on a short-term ward:

There I found my place. I’m very enthused about both of them (personnel)…It was what I needed at the time…Then, of course, it’s wonderful to get ready made dinner. And breakfast, have you seen our breakfast? It’s fantastic! (Woman, 87)

The joy of having meals that pleased both eye and palate made the daycare center a highly valued part of this participants’ everyday life. During the interview, she referred to the personnel by name several times while telling how they enrichened her day-to-day life. Another participant, having grown up in a rural area, emphasized the minibus trips to the daycare center, where they drove through parts of the rural area to pick up other attendants. After describing all the places they passed on the way, including the place where he grew up, he concluded, “When the weather is good, it’s a really nice trip!” (man, 87). Thus, although the participant enjoyed the daycare center as such, he also found a benefit outside the formal confines of the center, which would be further strengthened, in good weather. However, not all participants shared this positive view. A participant who had just started attending a daycare center called it her “after school program” and added,

The other day, when I was up there, they were going to throw a ball…Then I took a chair and sat down behind them, to be spared from having to throw a ball…Of course it’s a little fun as well, it is, but…I can understand that some may like it, but I don’t. Perhaps I’m a bit weird. (Woman, 75)

She had to admit that it was a little fun as well. Still, she experienced throwing a ball as something perhaps below her dignity, attacking her integrity. In addition, her dislike and unwillingness to participate in the activity also made her feel left out, leaving her wondering whether she was “weird” or not.

## Discussion

The participants shared a well of reflections on their experiences with the support measures in question, as well as attitudes toward future possibilities. To summarize these reflections, across all the measures explored, we found that the criteria for assessing the support measures in question did vary among the participants, often relying on small margins related to their degree of knowledge and understanding of the support measure questioned. Despite initial hesitations, in part ascribed to lacking knowledge or prejudice toward unfamiliar measures, the participants’ final judgments relied upon how the various measures might fit their individual needs and affect their everyday lives. A repeated finding within qualitative research, exploring the perspectives of persons with dementia, is an emphasis of sustaining autonomy and control over their own lives.^30–32^ Similarly, a review on the perceptions of older people in general, considering assistive technology found that being in control and individual adaptation of the technology was crucial.^33^

In a meta-ethnographic study exploring meaningful activities among persons with dementia, Han et al.^34^ describe how similar activities can meet different needs of various persons and, conversely, how dissimilar activities can serve to meet similar needs. This matches the descriptions provided by the participants of this study. When describing their satisfaction with these care and support measures, they emphasized different aspects of homecare and daycare centers. Negative experience or attitudes were seldom attributed to the support measures as such, but to specific aspects of these measures. E.g. beeping devices, the inability to handle equipment, antipathy toward certain activities or a volunteer who failed to meet expectations. Even though they pointed out the presumed benefits of differing measures, this did not outweigh their personal, subjective perceptions of detriment, as the following participant underscored: “I can understand that some may like it, but I don’t.” Strandenæs et al.^12^ similarly describes how daycare participants try not to require too much when it comes to activities.

Care philosopher Kari Martinsen describes different ways of seeing and meeting the other as an individual.^35^ She distinguishes between two ways of *seeing*—involving two different *eyes*. The one, which she calls *the recording eye*, is an eye that seeks to see individual traits, with intent view to systematize and classify the person, to be able to provide adequate support for individuals matching the specific classification. The woman no longer able to use her stove might illustrate some possible limitations of this recording eye. In this case, classified as a person with dementia, living alone, a stove guard had been installed to reduce fire hazard. The participant understood why the stove guard had been provided, but perceived it as a removal of her opportunity to uphold baking, a meaningful activity for her. Nygård^36^ describes how these easily accessible stove guards may be used as substitutes for more individualized care and support.

The other way of seeing is through the lens of what Martinsen^35^ calls *the double eye*. This way of seeing goes the reverse; rather than looking for individual traits to classify, one looks for common, recognizable traits in each single person. By taking this detour, one is able to perceive and recognize the individual particularities of the other. Consequently, Martinsen claims it is possible to perceive and meet the other as a whole and individual person and his or her personal appeal for help—detached from diagnosis or predetermined categories. Subsequently, healthcare professionals can see and recognize this appeal when emphasizing person-centeredness as described by Kitwood^37^ and seeing each individual living with dementia as an equal person. The female participant of this present study, who was personally invited to the daycare center may illustrate this; apparently, someone saw her need, contacted healthcare personnel at the daycare center, who in turn invited her to come there on a regular basis. When attending the daycare center, the personnel continued to be important persons in her day-to-day life. The other participant, praising the homecare service for their cheerfulness, may also exemplify this. Although the visits were short before they had to “fly off,” the smiles and simple questions of the healthcare personnel made her feel that she was seen, recognized, and taken care of. Thus, both these participants experienced meaningful relations in everyday life, an aspect which has been shown to be important for persons with dementia.^38^ For them, it was not the content of the measures as such that made the difference, but the relational qualities they enjoyed when interacting with their care providers. These examples may illustrate the simplicity as well as significance of establishing such relations.

Other examples do however illustrate how easily these relations may be disregarded, as in the case of the participant experiencing that the volunteer only “talked and talked and talked.” The talk was presumably well intentioned—but it was not what the woman expected; she expected to go for a walk. Martinsen^35^ describes how the recording eye, when focusing on tasks and problem solving, may make healthcare personnel and others blind to the persons’ actual needs—losing the ability to recognize and support individuality and personal preferences. Although assessing decision-making capacity may be complicated,^39^ Smebye et al.^17^ document how persons with dementia may participate in decision making in a variety of ways, given that the helper knows and understands the person, and is able to provide manageable choices. Therefore, they claim that the question should not be on whether or not the person may participate, but rather how to empower the person to participate in decision-making processes affecting his or her everyday life. Similarly, although some participants in this study had initial difficulties in following the conversation, when given time, space, and explanation, they were able to reflect and share, not only experiences but also their attitudes concerning hypothetical future needs. This might illustrate both the fault in denying patient participation based on superficial assessments and how simple adaptations might contribute to enable the person with dementia to make reflected judgments considering their own care.

The participants’ descriptions of what worked for them, and how it worked, along with their attitudes and personal preferences on relevant future support measures, reveal not only what works but also how this is related to individuality. Knowledge of the thin line between what persons with dementia experienced as supportive versus offensive, dignity preserving versus violating, may help us learn that adjusting support to fit the person are by far more in line with care philosophy^35^ than adjusting the person to fit the support measures.

### Implications for practice

This study shows that given time and space, home-dwelling persons with dementia may be able and willing to reflect on their experience and attitudes toward assistive technology, volunteer support, homecare services, and daycare centers. Their perceptions of how different measures might or might not fit their needs and personal preferences should therefore naturally be part of the discussion and decision-making process. Knowledge of the thin line between what persons with dementia experienced as supportive versus offensive, dignity preserving versus violating found in this study, may help healthcare personnel to naturally emphasize patient participation as a resource in adjusting support measures to fit each person living with dementia. As previously found by Tranvåg et al.,^40^ advocating the person’s autonomy and integrity is a primary foundation for dignity-preserving dementia care. By meeting people living with dementia with what care philosopher Martinsen calls a *double eye*, we suggest that healthcare personnel as well as family caregivers might be able to recognize and identify the individual personal appeal for care and support—beyond predetermined needs assumed for a “person with dementia.” In addition, in line with Kitwood,^37^ enabling the person cared for to come forth as a unique, autonomous person along these lines, will be a way of bringing theory of person-centered care into practice—ensuring their human right of retaining autonomy and integrity as well as participation in decision-making processes considering their own care and support.^21^

### Limitations

The study included a relatively homogeneous sample of persons with dementia attending daycare centers in one municipality. A more heterogeneous group could have brought other aspects to light. Conversely, knowledge of the participants’ specific diagnose or degree of dementia could have made the study more specific. To strengthen the study’s transferability, we have endeavored to provide thick descriptions and elaborated on the variance and peculiarity in the empirical data. Together with a thorough and repeated examination of the empirical data with constant reflection on our pre-understanding and searching for disconfirming evidence, we have striven to establish study trustworthiness.^41^

## Conclusion

This study explored the perceptions of persons with dementia of assistive technology, volunteer support, homecare services, and daycare centers. Given time, space, and explanation, the participants expressed a variety of experience and attitudes concerning these support measures. Their responses revealed that there might be a thin line between care and support experienced as supportive versus offensive, dignity preserving versus violating. Furthermore, this balance is based on individual preferences and perceptions of how the care and support might affect the individuals’ everyday life. This implies a need for increased attention in clinical care and future research, to develop and implement sound strategies for patient participation in decision-making processes concerning care and support for persons living with dementia.
